# The Cold-Bath Treatment of Typhoid Fever

**Published:** 1899-09

**Authors:** 


**The Cold-Bath Treatment of Typhoid Fever.** By F. E. Hare, M.D. Pp. xii., 196. London: Macmillan and Co., Limited. 1898.

It seems remarkable that the medical profession of this country should be charged with inertia, because the cold-bath treatment which has been in vogue for some thirty years should not be universally adopted. It is true, as Dr. Hare remarks, that the difficulties in the systematic adoption of the method have been greatly exaggerated; the plan of procedure which he describes (page 18) makes the whole thing easy for hospital wards, and there can be no reason why the bath should not be of far more general use if its absolute necessity is demonstrated. There are, however, other antipyretic methods, and it is only occasionally that the bath has to take precedence of these.

In a consecutive series of 1902 cases treated at the Brisbane Hospital in Queensland, Dr. Hare claims to have reduced the mortality from 14.8 per cent. in the years 1882 to 1886, to an average of 7.5 per cent. for the years 1887 to 1896, an improvement amounting practically to 50 per cent. This result harmonises with Osler's dictum: "The cold-bath treatment, rigidly enforced, appears to save from six to eight in each century of typhoid patients admitted to the care of the hospital physician." Any fallacies which tend to inhere in an enquiry of this sort are as far as possible minimised or eliminated, and it is argued by the author that the difference in mortality for the two periods is not due to any other accidental variations, but simply and entirely to the systematic use of cold bathing. We congratulate the author on his persistent and successful efforts.

